# ARID2 Chromatin Remodeler in Hepatocellular Carcinoma

**DOI:** 10.3390/cells9102152

**Published:** 2020-09-23

**Authors:** Robin Loesch, Linda Chenane, Sabine Colnot

**Affiliations:** 1 INSERM, Centre de Recherche des Cordeliers (CRC), Sorbonne Université, Université de Paris, F-75006 Paris, France; robin.loesch@inserm.fr (R.L.); chenane.linda.19@gmail.com (L.C.); 2 Equipe labellisée “Ligue Nationale Contre le Cancer”, F-75013 Paris, France

**Keywords:** ARID2, hepatocellular carcinoma, cancer, chromatin, SWI/SNF

## Abstract

Chromatin remodelers are found highly mutated in cancer including hepatocellular carcinoma. These mutations frequently occur in *ARID* (*AT-rich Interactive Domain*) genes, encoding subunits of the ATP-dependent SWI/SNF remodelers. The increasingly prevalent complexity that surrounds the functions and specificities of the highly modular BAF (BG1/BRM-associated factors) and PBAF (polybromo-associated BAF) complexes, including ARID1A/B or ARID2, is baffling. The involvement of the SWI/SNF complexes in diverse tissues and processes, and especially in the regulation of gene expression, multiplies the specific outcomes of specific gene alterations. A better understanding of the molecular consequences of specific mutations impairing chromatin remodelers is needed. In this review, we summarize what we know about the tumor-modulating properties of ARID2 in hepatocellular carcinoma.

## 1. Introduction to the SWI/SNF Chromatin Remodeling Complexes

Gene expression regulation is far from being a random phenomenon. It requires covalent modifications targeting both DNA via methylation, and histones via post-translational modifications (PTM), which shape the nucleosomes and control the DNA compaction into chromatin and the recruitment of specific transcription factors. The level of DNA compaction is highly dynamic and dictated by multiple and diverse histone PTM [1] and/or by the extent of nucleosome occupancy, which is modulated via a mechanism called nucleosome remodeling. ATP-dependent chromatin remodelers form large complexes are capable of physically remodeling the landscape of histones and factors on the DNA. Such complexes, like the BRG1/BRM-associated factors (BAF) and polybromo-associated BAF (PBAF) complexes (frequently referred to as the mammalian SWI/SNF complexes), have been widely implicated in transcriptional regulation and biological processes including development, differentiation, and cancer [2,3].

**Figure 1:**
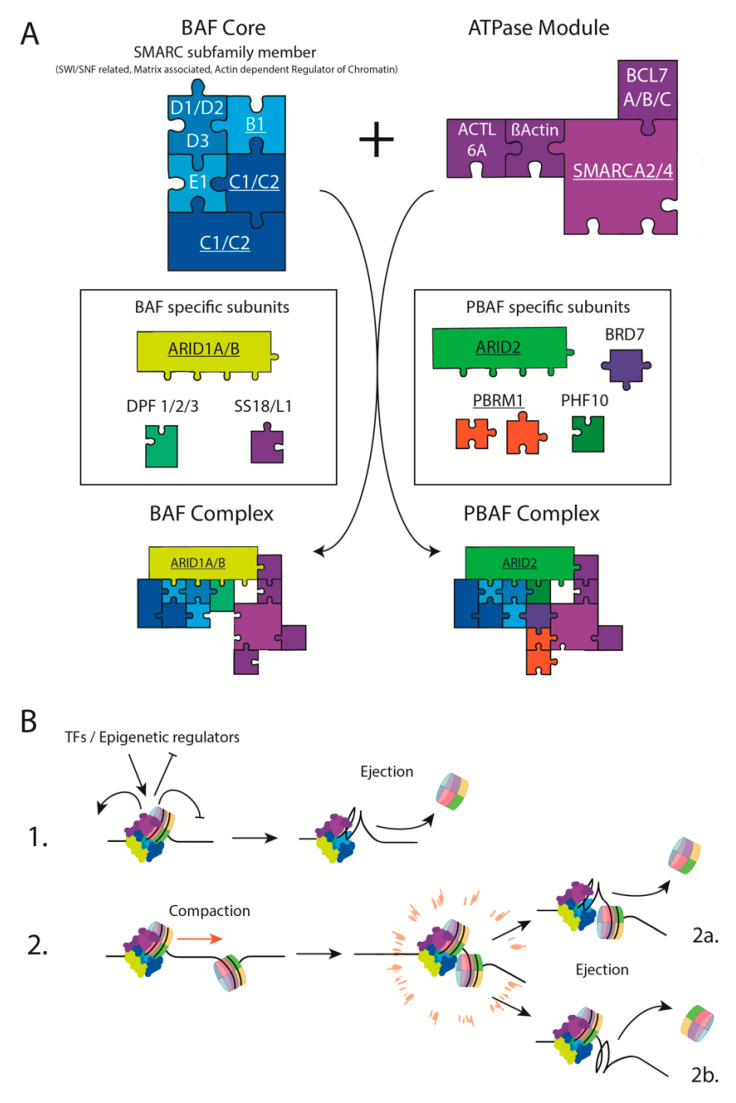
Structure and function of BAF/PBAF complexes. (**A**) Composition of the combinatory BAF and PBAF complexes. Multiple variant of the SMARC family members can be incorporated in the BAF core or the ATPase module. Addition of BAF and PBAF-specific subunits increases the combinatory potential of SWI/SNF complexes. Underlined subunits are found mutated in cancers. (**B**) Models of ATP-dependent SWI/SNF-mediated nucleosome remodeling. In the first model, SWI/SNF complex can eject nucleosome (**1**) while in the second one, DNA tension created by nucleosome sliding induces ejection of the adjacent (**2b**) or complex-bound (**2a**) nucleosome.

The identification of SWI/SNF subunits was a case of ultimate serendipity. Subunits were identified in two independent genetic screens in *Saccharomyces cerevisiae.* The first aimed to identify signal transduction molecules needed for the response to mating factors that lead to mating-type switching (SWI). The other screen was designed to identify signaling molecules leading to sucrose fermentation in response to nutrient switching in yeast (SNF; sucrose nonfermenting) [4,5]. The SWI/SNF components are related to the Trithorax family (TrxG), found in flies, to antagonize the polycomb group (PcG) proteins, in order to regulate Hox genes precisely [6]. The coevolution of the PcG and TrxG was accompanied by an increase in complexity. Mammalian PcG complex components are now separated into 3 complexes: the polycomb repressive complex 1 and 2 (PRC1/2) and the polycomb repressive deubiquitinase complex (PR-DUB). As for mammalian TrxG complex components, they are separated into the SWI/SNF and the COMPASS family. Mammalian SWI/SNF multiprotein complexes (Figure 1A) are now well described and appear, like PcG complexes, highly modular. BAF and PBAF SWI/SNF complexes are composed of 10 to 15 subunits, some of which are specific to the BAF (ARID1A/B; SS18/L1; DPF 1/2/3) or the PBAF (ARID2; BRD7; PBRM1; PHF10) complex. Shared subunits constitute the BAF core, composed of the SMARC subfamily members, and an ATPase module, containing the ATPase subunit BRM or BRG1 (SMARCA2 and SMARCA4 respectively). The precise mechanisms by which SWI/SNF complexes regulate gene expression are various and poorly understood. Early observations indicated that SWI/SNF complexes are capable of ATP-dependent nucleosome displacement and ejection which can be inhibited by histone mutants (Figure 1B) [7,8]. A recent study suggests different models by which the BAF complex can induce nucleosome ejection by itself and/or as a result of topological constraints [9]. Independently of nucleosome remodeling properties, SWI/SNF complexes could act as docking/recruitment platforms for epigenetic regulators as they have been shown to work alongside histone acetyltransferases (HATs) [10,11]. SWI/SNF complexes also notably appear to either antagonize polycomb-mediated repression or promote repression by polycomb in stem cells and cancer cells [3,12,13]. A study describing the role of the ATPase subunit in the composition and function of BAF and PBAF recently shed light on the regulation of those complexes. Pan et al. found that a residual BAF and PBAF complex can bind chromatin at overlapping sites in the absence of the ATPase subunit [14]. In this case, BAF and PBAF divergent localization on DNA was restored upon rescue of the ATPase subunit. Furthermore, a subset of binding sites are independent of the ATPase activity, supporting the idea that SWI/SNF functions are not necessarily linked to nucleosome remodeling. Moreover, this study supports the fact that the incorporation of specific subunits is potentially responsible for the SWI/SNF targeting.

Back in the late 1990s, the bi-allelic alteration of the SWI/SNF subunit SMARCB1 was first thought to contribute to oncogenesis in highly malignant childhood neoplasms [15]. The development of high-throughput sequencing technologies these last two decades has revealed more and more somatic mutations damaging SWI/SNF subunits in malignancies. Indeed, nearly fifty sequencing studies have revealed that several subunits of mammalian SWI/SNF are mutated in ~20% of the analyzed cancer types [2,16]. *ARID2* mutations were first identified in hepatocellular carcinoma (HCC) which suggested a tumor suppressive activity [17,18]. Here we review the current knowledge about the involvement of ARID2 in cancer in general and in HCC more specifically.

## 2. ARID2, Structure and Regulation

**Figure 2:**
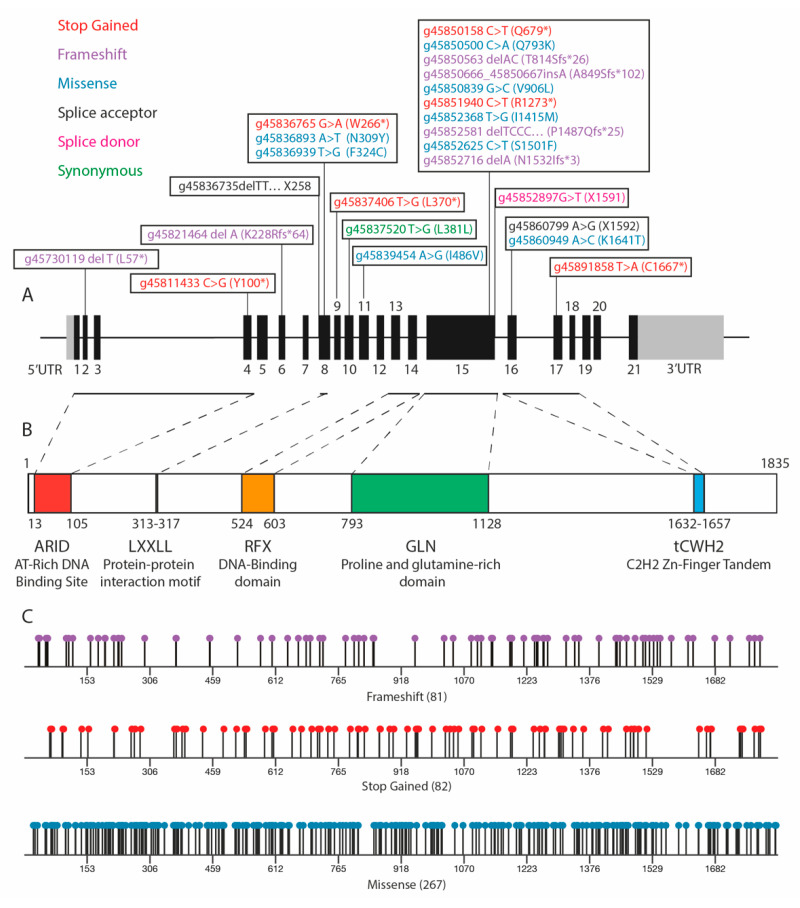
*ARID2* gene and protein, with gene mutations found in cancer. (**A**) *ARID2* gene structure along with mutations found in human hepatocellular carcinoma (HCC). (**B**) ARID2 protein structure with known domains. (**C**) Positions and nature of *ARID2* mutations found in cancer (pan-cancer, The Cancer Genome Atlas (TCGA) data).

ARID2, for AT-rich interactive domain 2, is a 1835 amino acid long protein encoded by *ARID2* gene on chromosome 12q (Figure 2B). ARID2 was first identified in a yeast two-hybrid screen as the Zipzap/p200 protein interacting with SRF (Serum Response Factor) [19]. In showing the activation potential of Zipzap/p200 on cardiac genes during development, a role for ARID2 as a transcriptional co-activator was proposed. Its name comes from the AT-rich DNA interaction domain (ARID) found in N-terminal which is common to the ARID protein family [20]. Like the other ARID members of the SWI/SNF complexes, ARID2 binds DNA without sequence specificity [21], but it is still unclear to what extent the ARID domain is important to the PBAF complex. Overall, the functionality of each ARID2 protein domain is still poorly known.

ARID2 contains two additional DNA-binding domains: an evolutionary conserved RFX domain [22] and a tandem C2H2 zinc finger domain, and one protein-protein interaction domain LXXLL. A recent study from Kadoch laboratory suggests that the core binding domain involved in the docking of ARID2 to the BAF core module extends from the ARID domain to the RFX domain [23]. A large proline and glutamine-rich domain is found in the middle of *ARID2*, but its function is still unknown. Such a domain is found in many transcription factors and might be important for protein-protein interaction. The conservation among mammals of the three DNA binding domains in ARID2 protein suggests a role in the binding of PBAF complex to the nucleosome-associated DNA. ARID2 was first shown to be the only subunit with a short half-life. ARID2 decreased expression, either by depletion using small interfering RNA [24] or CRISPR [25], leads to a diminution of other PBAF-specific subunits. It is in keeping with the recent description of the SWI/SNF complex assembly [23] that shows that ARID2 initiates PBAF assembly and is important for the recruitment and stability of PBAF-specific subunits.

## 3. ARID2 in Development and Pathophysiology

Related to its involvement in PBAF complex, ARID2 has been rapidly involved in the regulation of tissue-specific gene expression [26]. ARID2 is expressed in the majority of murine tissues, starting from embryonic stage E8, and is vital for normal embryonic development [27]. In vivo insertion of LacZ cDNA into *Arid2* murine locus, resulting in a hypomorphic *Arid2* mutant allele, reveals many heart impairments underlying the essential role of Arid2 in the proliferation of embryonic cardiomyocytes and their developmental fate. The conditional deletion of *ARID2* in endothelial cells and hematopoietic lineages using a Cre-lox approach in mice led to the reduction of the absolute cell number in fetal liver at mid-gestation (E13.5), severe anemia, growth delay starting from early E15.5, and embryonic death by E18.5 [28]. Moreover, Fuhua et al. used a shRNA approach to deplete *ARID2* expression in undifferentiated MC3T3-E1 pre-osteoblasts (committed to the osteoblast lineage) and found that ARID2 is required for osteogenic differentiation [26].

Consistent with early studies linking the SWI/SNF complexes to the expression of interferon responsive genes [29,30,31,32,33], Yan et al. [24] showed that knockdown of ARID2 in Hela and HCC1143 cells specifically inhibits the induction of a subset of interferon-induced genes such as *Ifitm1* (Interferon-Induced Transmembrane Protein 1).

PBAF complex and especially ARID2 were additionally reported to be involved in DNA damage response (DDR). In their study, Kakarougkas et al. [34] not only described the need for PBAF complex to silence transcription flanking DNA breaks, but also showed that PBAF acts jointly with PcG proteins to ensure efficient repair. Furthermore, they showed that PBRM1 phosphorylation by the serine/threonine kinase ATM is required for both repair and transcriptional silencing and cannot be rescued by a cancer-associated PBRM1 mutant. In another recent study showing the implication of ARID2 in DDR, de Castro et al. [35] showed that ARID2 interacts with RAD51, a protein involved in DNA repair of double strand breaks (DSB), and makes its recruitment easier into DSB sites upon repair via homologous recombination. Additionally, they showed that ARID2 and RAD51 interact via their C-terminal domain.

Lastly, in addition to cancers, some neurodevelopmental disorders have been linked to mutations in SWI/SNF complexes (BAFopathies) [36]. This is the case for de novo heterozygous *ARID2* mutations, which have been linked to the Coffin-Siris syndrome 6 (CSS6), a rare genetic intellectual disorder characterized by a mild to severe developmental or cognitive delay [37,38]. In CSS6, unique variants were found across the *ARID2* gene and predicted to disrupt ARID2 functions via frameshift or substitution. Four independent patients harboring either an ARID2 mutation at the beginning of the protein (2 of them) or next to the zinc finger motif, were found to have very similar CSS-like phenotypes which suggests that global heterozygous ARID2 loss-of-function (LoF) can cause CSS syndrome due to haploinsufficiency. However, as of now, no malignancies have yet been reported in CSS6 patients. This might be because a full loss of ARID2 is required for a tumorigenic effect or because ARID2 loss alone is not sufficient to promote oncogenesis.

## 4. ARID2 in Cancer

*ARID2* mutations have been reported in many human cancers including melanoma, urothelial cancer, gastric adenocarcinoma, non-small cell lung cancer, HCC, and more [16]. Sequencing data from The Cancer Genome Atlas (TCGA) do not reveal major mutation hotspots in *ARID2* and alterations are spread all along the gene in all cancers, including HCC (Figure 2A,C). Therefore, it is possible that the oncogenic features of mutated ARID2 are not mediated by the loss of a specific domain but rather by the global loss-of-function (LoF) of ARID2, most probably impacting PBAF functions. Several studies suggested or reported ARID2 tumor suppressor properties [18,39,40]. Exome data in melanoma showed a higher LoF mutation burden in *ARID2* than expected by chance [41] and nonsense mutations were predicted to lead to truncated proteins lacking the C2H2 zinc finger domain. *ARID2* was also found mutated in 5% of non-small cell lung carcinoma and, as for HCC, in a *TP53* non-mutated background [40].

While SWI/SNF complexes and ARID2 have been involved in both DDR and gene regulation, it is still not clear to what extent their tumor suppressive effect is due to impaired DDR or aberrant transcriptional regulation. However, there are some clues as to what might be happening. The recent description of PBAF complex assembly suggests that ARID2 or PBRM1 loss could lead to similar consequences. *PBRM1* germline mutations in clear cell renal cell carcinoma are not associated with increased chromosomal abnormalities or an increased mutational burden, nor are germline *SMARCB1* mutations in rhabdoid tumors, suggesting that SWI/SNF-mediated DDR is poorly or not involved in chromosomal stability in these cases. Another piece of information is that in vitro rescue of a functional SWI/SNF complex is frequently associated with a decrease in cellular proliferation and death [42,43]. If the loss of ARID2 or PBAF complex can impair DDR, an aberrant transcriptional regulation is more likely to be responsible for the tumor suppressive properties.

Finally, Arid2, together with Pbrm1 [44], is linked to cancer cell immunotherapy resistance [25]. Its expression inversely correlates with the expression of genes related to T cell cytotoxicity in several human cancers. Inactivation of Arid2 in B16-F10 melanoma cells makes them sensitive to T cell-mediated killing and enhances IFNγ response. ARID2-mutated cancers are therefore expected to offer a greater tumor susceptibility to immunotherapies.

## 5. ARID2 in Hepatocellular Carcinoma (HCC)

**Figure 3:**
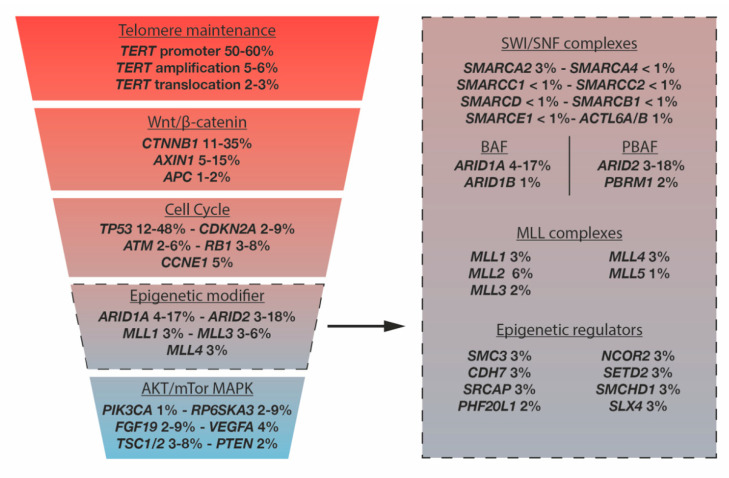
Hepatocellular carcinoma mutational landscape. The left part of the figure shows the different pathways affected by gene alterations in HCC, with the percentage of mutations; the right part shows mutations in epigenetic modifiers, the most frequently mutated being ARID1A and ARID2 (Adapted from Nault, J Hepatol 2014 [51]).

HCC is an example of cancer that frequently harbors *ARID2* mutations. HCC is the most frequent primary liver cancer for which patients are often diagnosed at advanced stages and only unsatisfactory treatment options are available. Although early-stage tumors can be cured using surgical approaches, less than 30–40% of HCC patients are eligible for such curative therapeutic options. For patients with unresectable HCC, multikinase inhibitors (TKI) such as sorafenib and lenvatinib are used to limit disease progression. A protocol has recently emerged combining TKI treatments with immunotherapies to better improve patient survival, giving hope to patients [45]. HCC is a highly heterogeneous cancer, frequently occurring in a context of cirrhosis, and related to etiologies including viral hepatitis C and B (HCV and HBV), alcohol intake, and non-alcoholic steatohepatitis [46]. Moreover, HCC development relies on a complex multistep process involving different genetic alterations and deregulated signaling pathways [46]. Recent high-throughput genomic analyses have converged on mutational hotspots in human HCC confirming telomerase expression, β-catenin signaling, TP53 related signaling, and chromatin modifications among the core deregulated pathways in HCC initiation and/or progression [47,48,49,50] (Figure 3).

### 5.1. Arid2 Suppressing Function in HCC

#### 5.1.1. Mutational Inactivation

Early exome sequencing studies investigated the mutational landscape in HCC, and *ARID2* inactivating mutations were the first to be identified among the SWI/SNF chromatin remodeler family in 2011 by Li and Zhao and colleagues [17,18]. Exon sequencing of 139 HCC found expected mutations in previously described genes like *CTNNB1* or *TP53* and unexpectedly LoF mutations in *ARID2. ARID2* alterations consist in frameshift-inducing insertion or deletion (indels), nonsense mutations, and splice-site mutations with no major hotspot of mutations (Figure 2A). In agreement with a more recent study [52], *ARID2* mutations were found correlated with *CTNNB1* mutations (in 6 out of 9 HCC) and mutually exclusive with *TP53* mutations. *ARID2* mutations were also found to occur more often in HCV-related HCCs (6/43) than in HBV (1/50) or non-viral (2/44) related HCC.

#### 5.1.2. MiRNA-Mediated Down-Regulation

MicroRNAs (miRNAs) are small noncoding RNAs which post-transcriptionally repress gene expression. Their widespread role in processes driving tumor initiation and progression is recognized [53]. Interestingly, multiple miRNAs (miR) were found to decrease ARID2 levels in HCC, thereby promoting tumor growth and invasion [54,55].

- MiR-208 was described to promote cell proliferation in human esophageal squamous cell carcinoma [56], promote EMT in pancreatic cancer cells [57], stimulate tumorigenesis in colorectal cancer [58], suppress cell apoptosis in gastric cancer [59], or to facilitate cell proliferation and invasion in non-small cell lung cancer and in hepatocellular carcinoma [54,60]. In 2015, miR-208-3p was found to be transcriptionally regulated by TGFß1 and to directly target ARID2 [54]. ARID2 downregulation, mediated by miR-208-3p, decreased proliferation and invasion in HepG2 and Hep3B hepatoma cell lines. Tumor growth was impaired after subcutaneous injection of Hep3B cells transfected with a miR-208-p3 inhibitor. In vitro, ARID2 restoration partially reversed the effect of miR-208-p3 inhibitor on proliferation and invasion. Altogether experimental data link TBFß signaling to ARID2 downregulation through miR-208-3p, leading to HCC cell proliferation and invasion.
- Mir-376c was described to be abnormally expressed, acting either as an oncomiR in ovarian cancer [61] and colorectal cancer [62] or as a tumor suppressor in melanoma [63]. Mir-376c was found to be upregulated in a screen for dysregulated miRNA in chronic HBV-associated HCC [64]. Further analysis found that miR-376c-3p expression promotes proliferation, migration, and invasion in Hep3B and SMMC-7721 cell lines through direct targeting of ARID2 via its 3′-UTR. As for miR-208, miR-376c-3p knockdown using an inhibitor restrained tumor growth in a xenograft mice model using Hep3B cells. In addition, miR-376c-3p expression was found to be significantly higher in HCC samples compared to non-tumor-adjacent tissues while ARID2 was inversely downregulated in HCC samples.
- Mir-155 is one of the most studied oncomiR that has an extensive role in malignant and non-malignant diseases [65]. Mir-155 appears to play multiple roles in HCC. Some evidence link miR-155 to (1) HCC cell proliferation via downregulation of PTEN [66,67]; (2) TGFß mediated epithelial-mesenchymal transition (EMT) [68]; (3) Wnt/ß-catenin signaling activation via downregulation of *Apc* during HCV infection [69]; (4) suppression of the tumor suppressor gene *FBXW7* [70]. Furthermore, two studies describe ARID2 as a direct target of miR-155 [71,72].

### 5.2. ARID2 is a Tumor-or Metastasis-Suppressor in HCC

In 2016, a lower expression of *ARID2* was globally found in tumoral tissue versus non-tumoral-adjacent tissue in 40 HCC samples, both at the mRNA and protein levels [73]. By overexpressing ARID2 in HCC cell lines (ARID2-Up), Duan et al. were able to suppress cell proliferation and migration in HCC cell lines, while downregulation of ARID2 by siRNA-mediated knockdown (ARID2-Kd) increased cell proliferation and migration capacities. ARID2-dependent proliferation was further confirmed in an orthotopic xenograft model using Arid2-Up or Arid2-Kd HCC cell lines. Using HCC cell lines with ectopic expression of E2F1, ARID2 downregulation was linked to Rb-E2F signaling and associated with transcriptional upregulation of E2F1, cyclins D1 and E1, CDK4, as well as Rb phosphorylation. ARID2 interacted with E2F1 and this was associated to cyclin D1/E1 repression via dissociation of E2F1 and RNA pol II from *CCND1* and *CCNE1* promoters. Duan et al. suggested a mechanism through which ARID2 controls cell proliferation by regulating the expression of cell cycle components. More recently, Jiang et al. described ARID2 downregulation in metastatic HCC, in association with poor prognoses [74]. ARID2-Kd was associated with increased migration and invasion in HCC cell lines whereas ARID2 over-expression in HCC cell lines and their related metastatic derivative inhibited invasion and migration. The suppressive effect of ARID2 on metastasis was further studied in vivo. When injected in mouse livers, the metastatic potential of HCC cell lines was reduced when ARID2 was overexpressed, while in contrast, this metastatic potential increased after ARID2 knockdown. Using a Cre-loxP mouse model of hepatic specific *Arid2* deletion (*Arid2*^ko^), in a context of chemically (DEN) or genetically (via TP53 deletion and Ras^G12D^ oncogene expression) induced carcinogenesis, the authors additionally demonstrated that ARID2 acts as a metastasis suppressor in HCC. ARID2 was found in HCC cell lines to downregulate mesenchymal markers such as Snail and upregulate epithelial markers such as E-cadherin. This suggested a suppressing effect of Arid2 on EMT which was supported by the induction of an EMT signature found in the transcriptome of primary *Arid2*^ko^ mouse hepatocytes. Focusing on Snail transcriptional factor, the authors described the binding of Arid2 on *Snail* promoter via interaction of C2H2 domain of Arid2 with Dnmt1. Further in vitro experiments supported a model in which Arid2 promotes Dnmt1 recruitment to *Snail* promoter, inducing promoter methylation and subsequent downregulation of *Snail* expression. Finally, the authors provided evidence that mutation of Arid2 C2H2 domain abolishes its metastasis suppressor function by losing its ability to recruit Dnmt1 to *Snail* promoter.

The loss of function of ARID2 was also shown to affect DNA repair in HCC. Regarding tumor initiation, one in vitro study [75] describes a role for ARID2 in recruiting XPG (xeroderma pigmentosum complementation group G), a component of the nucleotide excision repair (NER), upon UV irradiation or exposure to carcinogenic chemical compounds like benzo{a}pyrene and FeCl3. Such a defect in NER mechanism could induce an accumulation of somatic mutations leading to tumorigenesis in the liver, but it still needs further demonstration.

## 6. Towards Therapies against HCC by Targeting ARID2

**Figure 4:**
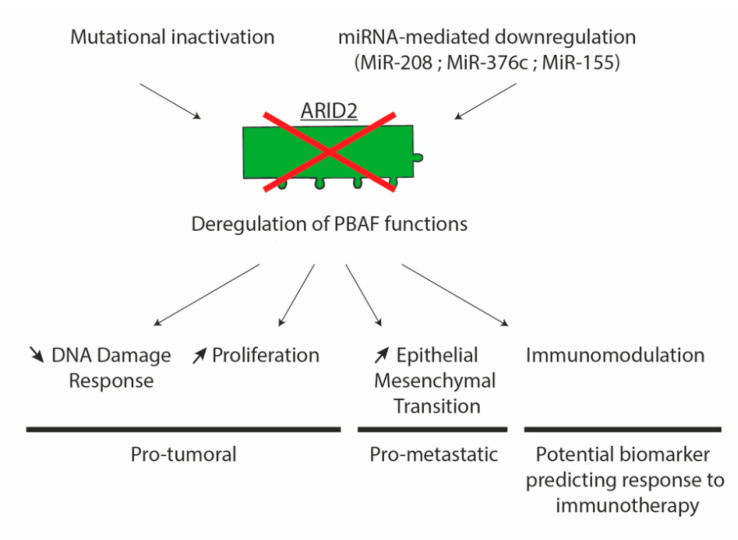
Potential impact of ARID2 loss in HCC.

*ARID2* is emerging as a tumor metastasis suppressor gene in HCC and cancer in general (Figure 4). For now, some underlying mechanisms have been described in liver cancer cells, such as ARID2 interaction with E2F1 to control the cell cycle or its interaction with Dnmt1 to control EMT and invasive/metastatic processes. SWI/SNF functions have already been found highly dependent on the tissue context. In this matter, it appears that mutations of SWI/SNF components can, most likely, induce very cancer-specific features. It also implies that appropriate in vivo models are needed to fully understand the tumor suppressive effect of SWI/SNF components.

In addition, oncogenic cooperation has still not been explored. In HCC, *ARID2* mutations have been associated with HCV infection and with ß-catenin activating mutations in *CTNNB1*. This can be correlated with the fact that *CTNNB1*-mutated HCC are mostly associated with non-viral and HCV etiologies [47,76]. A better understanding on how *ARID2* mutations could be elicited in a context of HCV infection, and/or how chromatin remodeling cooperates with Wnt/ß-catenin signaling in *ARID2* and *CTNNB1*-mutated HCC is required. Interestingly, this subset of HCCs forms a group independent of the *TP53*-mutated one, as it occurs in *ARID2*-mutated non-small cell lung carcinoma [40]. This mutual exclusion of *ARID2* and *TP53* mutations in cancer is to be further studied.

The involvement of *ARID2* mutations, and more widely the disruption of chromatin regulatory processes in cancer, point towards the need for anti-cancer therapies targeted on chromatin-associated proteins. With that in mind, genome-scale synthetic lethal studies have been undertaken in a wide spectrum of cancer model systems, using either RNA-interference or CRISPR-Cas9 editing [77]. They already identified dependencies specific to chromatin remodeling components and such an approach should be conducted in HCC models bearing alterations in chromatin remodelers like ARID2 [78]. In this matter, it is of interest to study the interaction between the different canonical SWI/SNF complexes (BAF and PBAF) as well as the recently described non-canonical BAF complex (ncBAF) [78] which has been described as free from any ARID subunit (ARID1A/B, ARID2). We can hypothesize that the loss of a subunit specific to one of the three complexes can alter the distribution and the amount of the others, thus explaining both the changes in gene expression and the dependency on other complexes for tumorigenesis.

Lastly, immunotherapy combined with TKI treatment is a promising therapeutic avenue for HCC. ARID2 has been recently thought of as modulating the tumor immune landscape in HCC [79]. Given the fact that the loss of ARID2 makes melanoma cells more sensitive to immunotherapies, it is now necessary to investigate the role of ARID2 as a biomarker predicting HCC response to immunotherapy.
